# Mobilising Knowledge in (and About) Academic Health Science Centres: Boundary Spanning, Inter-organisational Governance and Systems Thinking

**DOI:** 10.34172/ijhpm.2022.7314

**Published:** 2022-04-25

**Authors:** Alexandra Edelman, Robyn Clay-Williams, Michael Fischer, Roman Kislov, Alison Kitson, Ian McLoughlin, Helen Skouteris, Gillian Harvey

**Affiliations:** ^1^College of Public Health, Medical and Veterinary Sciences, James Cook University, Townsville, QLD, Australia.; ^2^Australian Institute of Health Innovation, Macquarie University, Sydney, NSW, Australia.; ^3^Melbourne Business School, University of Melbourne, Melbourne, VIC, Australia.; ^4^Manchester Metropolitan University, Manchester, UK.; ^5^The University of Manchester, Manchester, UK.; ^6^College of Nursing and Health Sciences, Flinders University, Adelaide, SA, Australia.; ^7^Formerly Monash Business School, Monash University, Melbourne, VIC, Australia.; ^8^Health and Social Care Unit, School of Public Health and Preventive Medicine, Monash University, Melbourne, VIC, Australia.; ^9^Caring Futures Institute, College of Nursing and Health Sciences, Flinders University, SA, Australia.

## Introduction

 Academic Health Science Centres (AHSCs), also termed Research Translation Centres (RTCs) in Australia, are organisations that aim to promote the integration of research, health professional education, and health service delivery to improve translation and innovation in Australia’s health system. In 2020, we published a study on how people, processes and systems were being organised within Australian AHSCs to enable knowledge to be mobilised for impact.^1^ The study, conducted in 2019, found that AHSCs in Australia were in an emergent state of development, following different pathways, and enacting varied strategies to support research translation. We argued that this presented an opportunity for formative learning and evaluation within the developing AHSCs to optimise their enactment of knowledge mobilisation (KM) processes for impact.^1^ In this correspondence, we reflect on three commentaries responding to the findings of our original paper.^2-4^ We consider the key issues raised by our colleagues with reference to our original study findings and the growing body of literature on AHSCs in Australia, highlighting: KM implementation challenges, the need for creative attention to governance, the need for systems-level KM approaches, and implications for AHSC evaluation.

## Knowledge Mobilisation Implementation Challenges: Co-production and Boundary Spanning Roles

 Jorm & Piper^3^ and Ferlie^4^ reflect that AHSCs exemplify policy interest in KM within Australia’s health system, with their organisational form influenced by “policy transfer” of the AHSC concept and characteristics from the United Kingdom and North America.^4^ A growing aspiration among policy-makers to drive “impact” within Australia’s health system indeed provides a compelling narrative behind the establishment and public funding for AHSCs in Australia.^5^ Yet, perhaps reflecting the emergent state of development of the AHSCs in our original study, we identified a need for more attention to KM processes, including investments in co-production processes and boundary spanning roles as key KM strategies.^1^ Our colleagues draw attention to some important practical challenges associated with these proposed strategies, which we highlight here.

 Ferlie^4^ describes AHSCs as organisations aspiring to drive “mode 2” knowledge production. Whereas “mode 1” represents traditional and academically directed approaches to research and translation, a defining characteristic of Mode 2 is its embrace of “co-production” via collaborative development and use of knowledge involving both researchers and end-users.^6^ Yet, as Ferlie^4^ notes, co-production in practice is challenging, requiring researchers to develop and apply specific, less traditional, skills. Jorm and Piper also describe a potential dissonance between what motivates university-based researchers compared with health service and policy practitioners, including the conundrum that co-production might not be palatable to researchers if the process drives overly specific, practice-focussed research with little generalisability or publication potential.^3,7^ To minimise the costs and maximise the benefits of co-production, Oliver et al^7^ call for researchers, funders, commissioners, and participants in the research process to address several key questions prior to attempting co-production, including: *What is everyone bringing to the table? Under which circumstances are co-production processes needed, for what purpose, and at which stage of the research process? What are the costs? How are decisions taken, and how will responsibility and accountability be shared?*

 Both Jorm and Piper^3^ and Spyridonidis^2^ also offer a critique of boundary spanning, or “knowledge broker,” roles as a solve-all KM strategy. Both authors posit that the introduction of individual knowledge broker roles into large, complex organisations is unlikely to generate sufficient momentum to address cultural barriers to organisational change. *“Studies highlight knowledge brokers merely generate a ‘ripple in the pond’ in a healthcare system consisting of tens of thousands of professionals, and a myriad of health and social care organizations.”*^2^ Exploring the “dark side” of knowledge brokering, Kislov et al^8^ call for organisational investment in multi-dimensional skillsets and a shift away from individual “brokers” towards collective brokering processes supported at organisational and policy levels. McLoughlin et al^9^ show in the Australian context that the absence of such supported processes makes new knowledge difficult to spread and scale, even where brokering efforts are sustained over several years.

## Inter-organisational Collaboration and the Need for “Creative Attention to Governance”

 Jorm and Piper^3^ draw a link between multi-organisational collaboration through AHSCs and their capacity to deliver effective KM: *“The RTCs [AHSCs] themselves are essentially brokering structures, and if they flourish, designing and undertaking substantive amounts of genuinely collaborative work, KM will follow.”* Although this expresses a widespread aspiration of AHSC leaders, we caution against giving the false impression to policy-makers and practitioners that high-level cooperation in the form of joint pursuit of agreed goals will automatically drive KM practices in AHSCs, without attention to concurrent coordination mechanisms in the form of alignment of organisational and administrative actions. In our original study, we identified a need for more attention in AHSCs to mechanisms of both cooperation and coordination,^1^ which are key tenets of multi-organisational collaboration.^10^

 We draw attention to the growing body of evidence on the limitations of inter-organisational governance approaches currently pursued in AHSCs. In a multi-country study on AHSCs in Australia and the United Kingdom, Robinson et al^11^ found that “dissonant metrics and drivers for healthcare improvement and research” between partnering organisations in AHSCs detracted from efforts to drive collaborative, impactful research. Collaboration challenges arising from bifurcating accountabilities between separate organisational partners in multi-organisational AHSCs in England are also reported by Ovseiko et al^12^ In their commentary, Ferlie^4^ describes AHSCs in the United Kingdom as “confederations rather than a single vertically integrated organization,” recognising the competing priorities and incentives this produces within multi-organisational AHSCs. As Ferlie describes of the UK context: *“The partners (National Health Service [NHS] Trusts and Universities) retain their own Boards and governance systems, so that the organization of the AHSC here takes the form of a relatively small cross organizational team laid on top of large and complex sovereign organizations.”*^4^ Thus, Ferlie^4^ underscores the powerful incentives underpinning the current separation of academic work and health service delivery; which are also recognised by Jorm and Piper^3^ and highlighted in our original study.^1^

 To address these challenges, Jorm and Piper^3^ call for “creative attention to governance” and greater involvement of the health sector partners through control of funding; yet precisely what governance structures and mechanisms might be best suited to diverse AHSCs in Australia remains unknown. We support calls to strengthen governance and accountability mechanisms in AHSCs, which is likely to require attention to power dynamics between AHSC organisational partners that demonstrate different budget sizes, regulatory mandates, and geographic and demographic foci. We suggest that future research to explore governance mechanisms consider the key questions posed by Ferlie: *“are these governance arrangements always cooperative in practice or are there some underlying tensions, given the scale and importance of these confederations? How is legal liability distributed in these complex settings? Does the overall AHSC Board have real powers or do these remain with the Boards of the constituent organizations which may remain legally sovereign? How is budgetary authority constituted and who is financially liable if the AHSC goes into financial crisis?”*^4^

## The Need for Systems-Level Knowledge Mobilisation Approaches

 The KM literature is often focussed on clinical contexts and mechanisms to drive improvements to healthcare; yet there is growing awareness of the need for KM science to address systems-level issues and improve public policy.^13^ Our original study demonstrated that a wide range of KM strategies are likely to be needed to support achievement of the varied academic, clinical, policy and population impact aspirations of AHSCs in Australia.^1^ Ferlie^4^ suggests that the rural, regional and remote focus of some Australian AHSCs is a key point of difference compared with UK AHSCs, which tend to have a metropolitan focus and a general orientation towards basic research and hospital settings. The inclusion of primary and community healthcare organisations as partners in several Australian AHSCs therefore offers a unique opportunity to develop and trial KM approaches that target systems-level imperatives such as addressing population health inequities.^14^

 Recent theoretical work in the KM field highlights systems approaches to KM practices. Haynes et al^13^ describe a key characteristic of “systems thinking” KM practices as a pluralistic view of knowledge, with mobilisation enacted through “continual dialogue” with policy and practice contexts to address intractable health systems challenges. Both Ferlie^4^ and Jorm and Piper^3^ draw attention to the contested nature of knowledge and the ways in which different types of knowledge come to be prioritised. Reflecting on the community and population health aspirations apparent in two of the AHSCs in our original study,^1^ Ferlie^4^ queries the implications of these contextual characteristics for the types of knowledge that might be valued, and in turn KM processes that might be pursued in these AHSCs. We support the need for further research to address the questions posed by Ferlie: “*What does high quality research-based knowledge mean here? What designs, methods and data sources are used in such research and are they different from the emphasis on Randomised Control Trials often apparent in the acute sector? Do primary, community and social care-based forms of evidence here have a higher profile? How evident is public health and population-based knowledge and evidence? Furthermore, how can a ‘good’ research knowledge base be mobilised effectively in these more geographically distributed settings with fewer large core institutions which might be expected to have some internal knowledge processing capacity?”*^4^

## Implications for Evaluation of Academic Health Science Centres

 Our original paper,^1^ and our commenting colleagues,^2-4^ all report a need for strengthened evaluation of AHSCs, noting the likely benefits to the AHSCs, and for broader accountability purposes, of both formative evaluation and longer-term impact assessment. Identifying appropriate impact indicators on which to base this evaluative work will be critical. Jorm and Piper^3^ argue that impact indicators should incentivise impact relevant to the specific contexts and priorities of each AHSC: *“Local impact is the major RTC outcome we should be seeking.”* Ferlie^4^ queries whether commercialisation should be a focus. Both local and system-level impact indicators are likely to be important to achieve a balance between context-specific priorities and cross-AHSC benchmarking and improvement. We argue that the very process of developing evaluation frameworks will help AHSCs to clarify their impact aspirations and set a foundation for the development of appropriate KM strategies to drive healthcare and health systems impact through effective collaboration.

## Acknowledgements

 The authors thank the experts who developed commentaries on our original study for their important insights and reflections. The authors also thank the interviewees from the participating AHSCs in the original study who generously offered their time for the research.

## Ethical issues

 Ethics approval for the original study on which this correspondence is based was obtained from the University of Adelaide Human Research Ethics Committee (H-2018-239).

## Competing interests

 Authors declare that they have no competing interests.

## Authors’ contributions

 AE produced the first draft of the manuscript. GH, RCW, MF, RK, AK, IM, and HS provided critical feedback on the manuscript draft.

## Disclaimer

 RK is partially funded by the National Institute for Health Research Applied Research Collaboration (NIHR ARC) Greater Manchester. The views expressed in this publication are those of the authors and not necessarily those of the National Institute for Health Research or the Department of Health and Social Care.
